# Pathological findings in a Dachshund-cross dog with neuroaxonal dystrophy

**DOI:** 10.1186/s13028-016-0218-3

**Published:** 2016-06-07

**Authors:** Davide Pintus, Maria Giovanna Cancedda, Simona Macciocu, Claudia Contu, Ciriaco Ligios

**Affiliations:** Istituto Zooprofilattico Sperimentale della Sardegna “G.Pegreffi”, Via Duca degli Abruzzi 8, 07100 Sassari, Italy

**Keywords:** Dog, Brain, Proprioceptive, Neuroaxonal dystrophy

## Abstract

**Background:**

Neuroaxonal dystrophy (NAD) is a neurodegenerative condition affecting humans and animals characterized by the widespread presence of swollen axons (spheroids).

**Case presentation:**

Herein, we report the pathological findings in a case of neuroaxonal dystrophy in a Dachshund-cross puppy, which was euthanized because of a proprioceptive positioning deficits and irreversible ataxia of the hind limbs. Histologically, there was a bilaterally symmetric neuroaxonal dystrophy with eosinophilic axonal spheroids exclusively localized at the level of the ventral posterior lateral nucleus of the thalamus, medial lemniscus, gracilis nucleus, medial cuneatus nucleus in the brain as well as the gracilis and cuneatus fasciculi throughout the spinal cord.

**Conclusion:**

To the authors’ knowledge, this is the first report of canine neuroaxonal dystrophy with this exclusive and specific localization only in the neuronal circuit implicated in the transmission of conscious proprioceptive information.

## Background

Neuroaxonal dystrophy (NAD) describes a group of inherited or acquired neurodegenerative conditions affecting humans and animals characterized by the widespread presence of swollen axons (spheroids) in the central and, rarely, peripheral nervous system.

Axonal spheroids in NAD affected dogs [1] and cats [2] have often been found in association with other changes such as cerebellar atrophy displaying marked loss of Purkinje cells [3, 4].

Several reports have suggested that NAD is an autosomal recessive disorder and some dog breeds have a recognized predisposition to NAD [5, 6]. Rottweilers are the most frequently affected breed [3], although NAD has been described in Jack Russel Terriers [7], Collie Sheep dogs [8], Chihuahuas [9], and Papillon dogs [10]. Among other species, few cases of NAD have been reported in sheep [11], cats [2] and raccoons [12], while in human, Hallervorden-Spatz syndrome and Seitelberger’s disease (INAD) share pathological hallmarks with the NADs observed in companion animals [7]. As an acquired condition, NAD can also occur in chronic vitamin E deficiency, insulin-deficient diabetes and exposure to certain toxins [13, 14].

The mechanisms by which spheroids develop in NAD are only partly known, but immunohistochemically methods have shown that NAD entails an accumulation of synapse-associated proteins, cytoskeletal proteins and other axonal markers, thus suggesting an axonal transport deficit as the underlying cause of spheroid formation [15].

Herein, we describe a case of NAD in a Dachshund-cross breed puppy with exclusive and specific neuro-distribution throughout the neuronal circuit implicated in the transmission of conscious proprioceptive information.

## Case presentation

Two Dachshund-cross breed puppies belonging to the same litter developed progressive difficult walking since they were a few weeks old. One puppy was euthanized without post mortem examination, while the other puppy was presented at 1 year of age for clinical examination. Neurological examination reveled hypermetria, proprioceptive positioning deficits and irreversible ataxia, particularly of the hind limbs. Muscular tone, bulk and strength were normal and higher mental functions appeared integral. The dog was humanely euthanatized because of the progressive incurable nature of the disease.

At necropsy, brain, spinal cord, tracts of the sciatic nerve, as well as samples from a number of forelimb muscles, liver, and kidney were collected and promptly fixed in 10 % neutral buffered formalin. Coronal sections were obtained at six different levels of the encephalon (basal nuclei, thalamus, mesencephalon, pons, medulla oblongata, cerebellum), as well as at the levels of each emergence of the nerves of the spinal cord and then routinely processed and embedded in paraffin wax. Sections were cut serially from paraffin blocks at 5 µm and stained using hematoxylin and eosin, modified Period Acid Shiff Picro Indigocarmine Morel–Maronger (PAS-M.M.), Klüver-Barrera Luxol fast Blue (KB), and Perl’s stain. Furthermore, in-depth immunohistochemical investigations were performed on selected tissue sections using specific primary antibodies for the following markers of axonal transport: neurofilaments light chain (NF-Ls,) and tau, cytoskeletal proteins; ubiquitin, a heat shock protein; synaptophysin, a synapse-associated protein; glial fibrillary acidic protein (GFAP), the specific marker of astrocytes (Table 1). To investigate the possible role of autophagic mechanisms in the formation of spheroids, a specific primary antibody against the autophagosomal microtubule associated protein 1A/1B-light chain 3 B (LC3B) was used (Table 1). Primary antibodies were always incubated overnight at 4 °C and immunoreactivity (IR) was detected via the biotin-avidin-peroxidase method, using the 3,3′-diaminobenzidine as a chromogen, Additional technical details are shown in Table 1. Transversal and longitudinal sections of the sciatic nerve as well as sections from muscles, liver and kidney were also embedded in paraffin, cut serially from the paraffin blocks at 5 μm and stained using hematoxylin and eosin. Finally, sections of the sciatic nerve were also stained immunohistochemically for GFAP, and histochemically for myelin by using KB technique. Immunohistochemically negative controls were obtained by omitting the primary antibody and using normal brain tissues of two crossbreed dogs aged 6 months and 1 year, respectively.

**Table 1:** Technical details of the immunohistochemical examinations

Primary antibody	Host	Dilution	Antigen retrieval	Manufacturer (code no)
Neurofilaments (NF-L)	Mouse	1:800	Citrate buffer 0.01 M pH 6.0 (3 × 5 min at 750 W)	Dako, Carpinteria, CA (M 0762)
Synaptophysin	Mouse	1:25	Citrate buffer 0.01 M pH 6.0 (3 × 5 min at 750 W)	Dako, Carpinteria, CA (M 0776)
Ubiquitin	Rabbit	1:150	None	Dako, Carpinteria, CA (Z0458)
Tau	Rabbit	1:100	Citrate buffer 0.01 M pH 6.0 (3 × 5 min at 750 W)	Sigma-Aldrich, St. Louis, MO (T5530)
Glial fibrillary acidic protein (GFAP)	Rabbit	1:100	None	BIOGENEX, Freemont, CA (932-020 M-4)
LC3B	Rabbit	1:800	Citrate buffer 0.01 M pH 6.0 (3 × 5 min at 750 W)	Abcam, Cambridge, UK (Ab 48394)

**Fig. 1:**
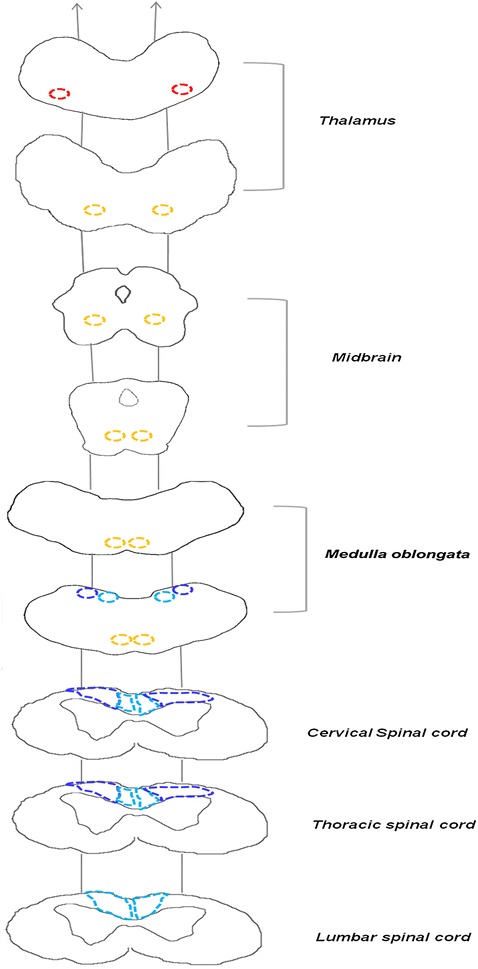
Distribution of the axonal spheroids. Illustration of the encephalic and spinal sections indicating the fasciculi and nuclei with spheroids throughout the central nervous system. Brain section: *Red dotted circle*: ventral posterior lateral nucleus of the thalamus; *yellow dotted circle*: medial lemniscus; *turquoise dotted circle*: gracilis nucleus; *blue dotted circle*: medial cuneatus nucleus. Spinal cord sections: *turquoise dotted circle*: funiculis gracilis; *blue dotted circle*: funiculis cuneatus

**Fig. 2:**
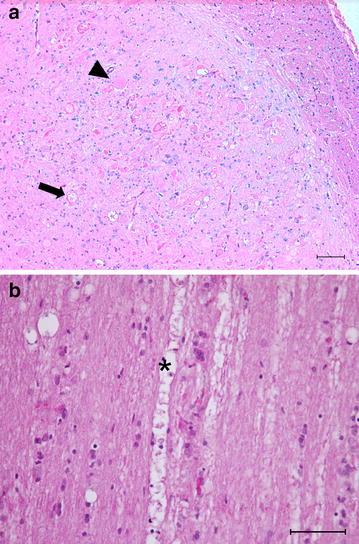
Photomicrographs of the histological patterns. Section of the medulla oblongata showing the medial cuneatus nuclei (**a**). Presence of numerous 3–50 µm sized eosinophilic axonal spheroids (*arrow head*), some of them are vacuolized (*arrow*). Thoracic tract the spinal cord (**b**). Longitudinal section at level of gracilis and cuneatus fasciculi displays digestion chambers with axonal debris and scattered macrophages (*asterisk*). HE. *Bar* = 100 µm

At necroscopy, no gross changes were observed. Histological examination throughout the central nervous system (CNS) revealed a bilateral symmetric neuroaxonal dystrophy, which was uniquely at the level of the ventral posterior lateral nucleus of the thalamus, medial lemniscus, gracilis nucleus, and medial cuneatus nucleus in the brain. The same change in the spinal cord was found in the gracilis and cuneatus fasciculi, particularly in the thoracic tract (Fig. 1). In the transversal sections, neuroaxonal changes were characterized by 3–50 µm sized axonal spheroids, round to ovoid in shape and stained slight to intensely eosinophilic (Fig. 2a).

In longitudinal sections at the level of the gracilis and cuneatus fasciculi, aspects of Wallerian-like degeneration with different digestion chambers containing sporadic macrophage and axonal debris were found (Fig. 2b). Occasionally, spheroids were found in the dorsal horn of the spinal cord.

Histochemical staining revealed that some spheroids contained PAS-M.M positive material and KB staining evidenced relative preserved myelin. Perl’s stain did not highlight the presence of iron in any sections of the encephalon.

**Fig. 3:**
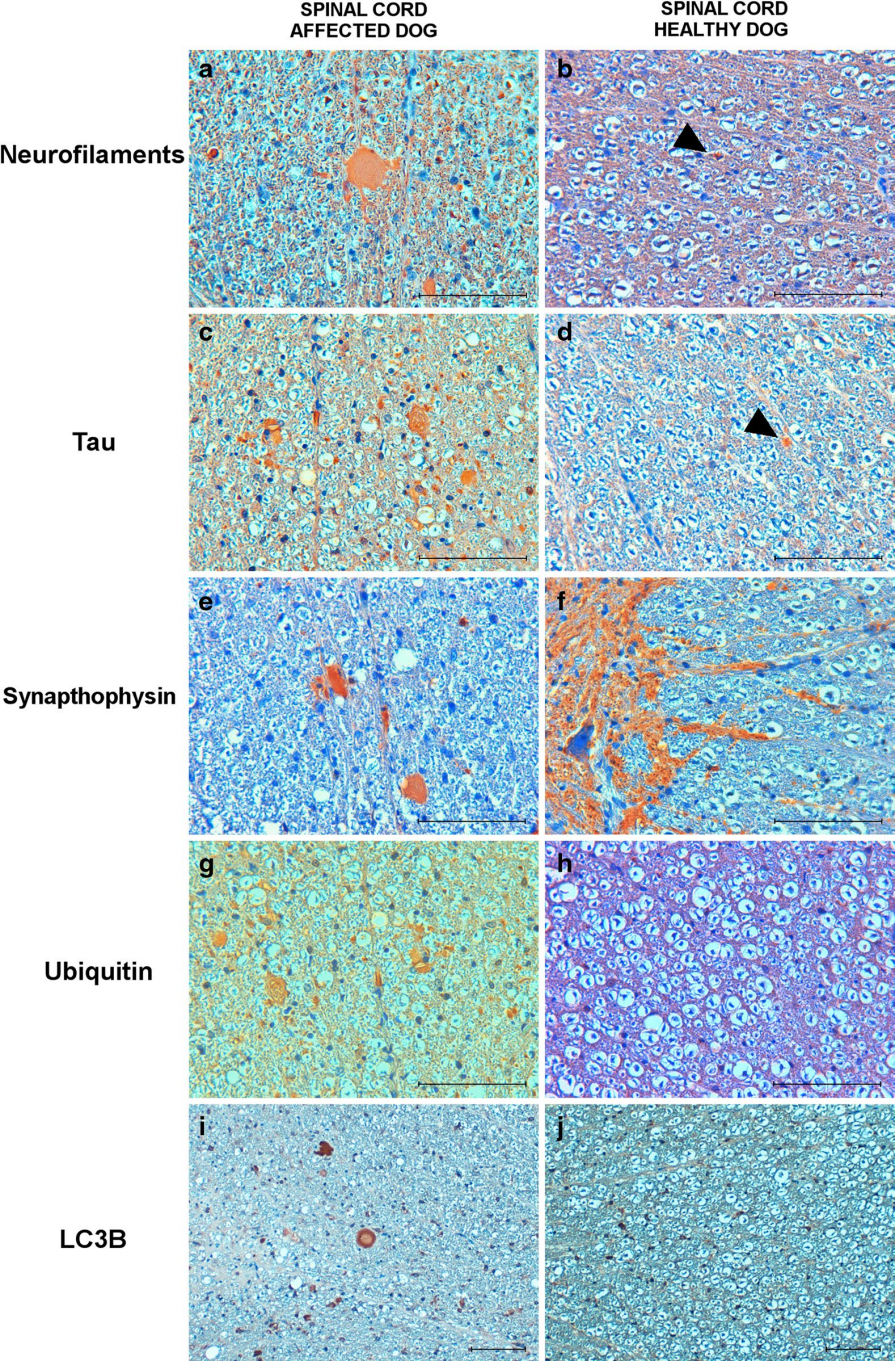
Photomicrographs showing immunohistochemical reactivity of axonal spheroids. Transversal sections of thoracic spinal cord (gracilis and cuneatus fasciculi) show numerous spheroid immunohistochemically reactive (IR) for neurofilaments (**a**), tau (**c**), synaptophysin (**e**), ubiquitin (**g**), and LC3B (**i**) of thoracic tract of the spinal cord the NAD-affected puppy. As expected, single spheroids were identified in the healthy dog (**b**, **d** *arrow head*), while LC3B IR is observed in the glial cells of the affected and control dogs (**i**, **j**). Synapthophysin was also evident in the neuropil of the control (**f**). Avidin–biotin-peroxidase complex method with Mayer’s hematoxylin counterstain. *Bar* = 100 µm

In all CNS sections, spheroids showed immunoreactivity for NF-Ls, tau, synaptophysin and ubiquitin with the signal being more intense at the level of the spinal cord (Fig. 3). In addition, immunohistochemistry for LC3B protein detected an intense autophagosome accumulation within spheroids (Fig. 3).

**Fig. 4:**
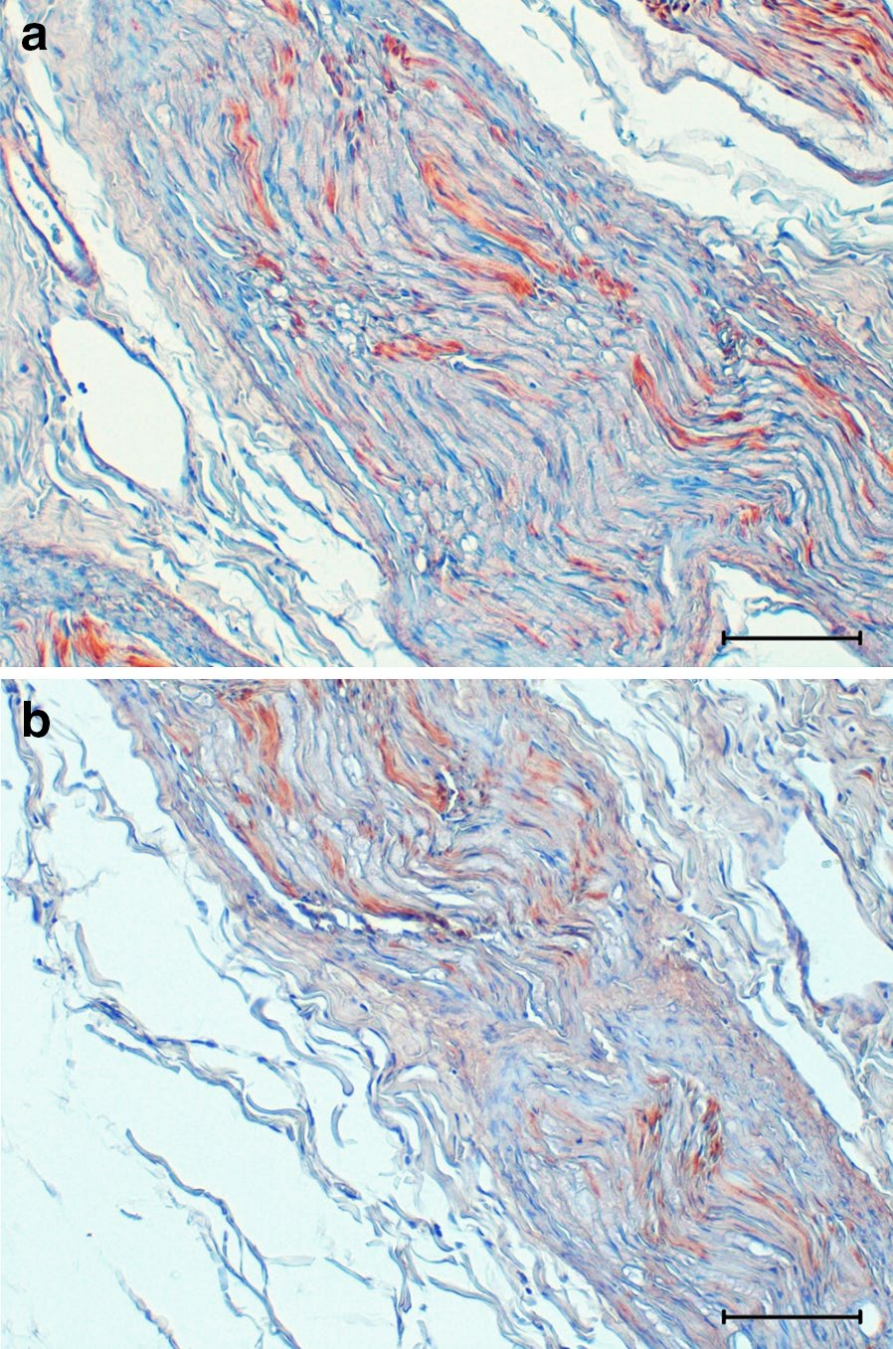
Photomicrographs of the immunohistochemical reactivity of the sciatic nerve. Sciatic nerve from NAD-affected dog (**a**) and healthy control dog (**b**). Longitudinal section of sciatic nerve shows weak linear immunohistochemical reactivity for GFAP both in affected (**a**) and healthy dog (**b**). Avidin–biotin-peroxidase complex method with Mayer’s hematoxylin counterstain. *Bar* = 100 µm

Finally, the immunohistochemistry for GFAP confirmed the presence of evident astrogliosis and astrocytosis in the nuclei displaying spheroids. The sections from muscles, liver, kidney and sciatic nerve were histologically normal. As in the control, the affected dog showed weak GFAP immunoreactivity with a linear pattern in the longitudinal sections of the sciatic nerve (Fig. 4). In the same nerve, no demyelization was observed by using KB stain.

On the basis of these clinical, histopathological and immunohistochemical features, a NAD disorder was diagnosed.

## Conclusions

In NAD, distribution patterns of the spheroids differ and involve numerous areas of the grey and white matters of the brain and spinal cord with the nucleus gracilis and nucleus cuneatus along with the dorsal horn of the spinal cord being constantly and predominantly affected [3].

So far, in Papillon [10], Rottweiler [3, 16] and Jack Russel Terrier puppies [7], the NAD lesions have been reported as widespread and distributed throughout the CNS. Interestingly, in Chihuahua puppies spheroids were mainly detected in the white matter of the brain [9]. With regard to the spinal cord, the distribution of spheroids has generally been observed in all the spinal funiculi of the cervical, thoracic and lumbar tracts of the spinal cord in canine NAD cases [4, 9]. Other changes such as a decreased number of cerebellar Purkinje cells [16]. Collectively, these reports indicate that, although axonal spheroids in NAD have been localized in a large number of nuclei and fasciculi, a distinctive involvement restricted to only one specific nervous circuits has not been reported yet. In contrast, the main feature of our case is the exclusive and specific localization in the neuronal circuit implicated in the transmission of the conscious proprioceptive information.

Previous reports of canine NAD have reveled immunopositive reaction in spheroids for several synapse-associated proteins, including synaptophysin, together with other molecules such NF-Ls, tau and ubiquitin [15, 17]. These observations indicate the presence of aberrant localization of integral synaptic vesicles, synaptic vesicle-associated presynaptic plasma membranes, and cytosolic proteins involved in the vesicular neurotransmitter transporter trafficking [15–17]. Regarding immunohistochemistry for LC3B protein, our results confirm that disturbance of the neuronal autophagy pathway is involved in the pathogenesis of spheroid formation. Recently, this has been demonstrated in NAD affected Spanish water dogs in which the disease was associated with a mutation of *tectonin beta*-*propeller repeat*-*containing protein 2* (*TECPR2*) gene [6], which is involved in the autophagic pathways, axonal transport, and mitochondrial metabolism.

Iron accumulation, within and outside the spheroids, is a characteristic aspect of human cases of the Hallervorden-Spatz syndrome. However, neuronal accumulation of iron was neither found in our case nor reported in other domestic animal NAD cases [15] thus excluding a Hallervorden-Spatz syndrome-like animal counterpart.

The immunohistochemical findings reported in the present NAD case are largely in agreement with those reported in dogs by other researchers; nevertheless, the distribution of the lesions in the present case remains dissimilar from most of the neuroaxonal dystrophies reported previously.

This dissemination of axonal spheroids has been documented in other species as well. In cats, vacuolization and spheroids were described in the fasciculus gracilis and fasciculus cuneatus, but also the dorso-lateral funiculi and the ventro and ventro-medial funiculi of the spinal cord were mildly affected [2].

Axonal dystrophy in NAD has until now been thought to display a wide distribution in the CNS involving different neuronal pathways and not exclusively the sensitive ones. Our case seems to be a topographically distinctive pathological entity characterized by lesions exclusively to sensory axons controlling general conscious proprioceptive information, which travels from the mechanoreceptors to the primary somatic sensory cerebral cortex throughout the fasciculus gracilis and cuneatus, gracilis and medial cuneatus nuclei, medial lemniscus, and ventral posterior lateral nucleus of the thalamus. In fact, the affected puppy’s clinical neurological symptoms mirror and are consistent with the location of these observed lesions. The dorsal column carries the majority of information of tactile discrimination and proprioception and terminates by contacting in the gracilis and medial cuneatus nuclei. Lesions of the dorsal columns of the spinal cord in primates hamper the ability to perceive the tactile stimuli and degrade the capacity to sense the position of the limbs in a given space [18]. In our case, the histological and immunohistochemical examinations of the sciatic nerve demonstrated there was no involvement of the peripheral nervous system. In fact, GFAP is overexpressed in Schwann cells of the nerves, once they are damaged. [19].

Although no proof of inheritance was determined in the present NAD case, a genetic disorder may be speculated, because of the congenital derivation and the presence of one other affected puppy in the same litter. Interestingly, our case is also the first to be described in a cross-breed dog. Finally, our report description stimulates the need for more systematic efforts to define and accurately describe all the clinical and pathologic manifestations of NAD. The literature illustrates that a wide range of pathological findings, which vary greatly among different species and breeds and from case to case, are all commonly categorized as NAD. It would be beneficial to invest in more careful and systematic post-mortem examination of the CNS in order to define NAD subtypes based on lesion location and corresponding neurological symptomatology.

## Abbreviations

CNS: central nervous system
GFAP: glial fibrillary acidic protein
INAD: Seitelberger’s disease
KB: Klüver-Barrera Luxol fast Blue
LC3B: autophagosomal microtubule associated protein 1A/1B-light chain 3 B
NAD: neuroaxonal dystrophy
NF-Ls: neurofilaments
PAS-M.M.: Period Acid Shiff Picro Indigocarmine Morel–Maronger
